# Long-Term Polygraphic Monitoring through MEMS and Charge Transfer for Low-Power Wearable Applications

**DOI:** 10.3390/s22072566

**Published:** 2022-03-27

**Authors:** Alessandro Manoni, Alessandro Gumiero, Alessandro Zampogna, Chiara Ciarlo, Lorenzo Panetta, Antonio Suppa, Luigi Della Torre, Fernanda Irrera

**Affiliations:** 1 Department of Information Engineering, Electronics, and Telecommunications, Sapienza University of Rome, 00184 Rome, Italy; 25chiara94@gmail.com (C.C.); lorenzo.panetta92@hotmail.it (L.P.); fernanda.irrera@uniroma1.it (F.I.); 2 STMicroelectronics, 20864 Agrate Brianza, Italy; alessandro.gumiero@st.com (A.G.); luigi.dellatorre@st.com (L.D.T.); 3 Department of Human Neurosciences, Sapienza University of Rome, 00185 Rome, Italy; alessandro.zampogna@uniroma1.it (A.Z.); antonio.suppa@uniroma1.it (A.S.); 4 IRCCS Neuromed, 86077 Pozzilli, Italy

**Keywords:** wearable sensors, long time domestic monitoring, multiple biopotentials acquisition, electrostatic sensors, low power consumption, MEMS technology

## Abstract

In this work, we propose a wireless wearable system for the acquisition of multiple biopotentials through charge transfer electrostatic sensors realized in MEMS technology. The system is designed for low power consumption and low invasiveness, and thus candidates for long-time monitoring in free-living conditions, with data recording on an SD or wireless transmission to an external elaborator. Thanks to the wide horizon of applications, research is very active in this field, and in the last few years, some devices have been introduced on the market. The main problem with those devices is that their operation is time-limited, so they do not match the growing demand for long monitoring, which is a must-have feature in diagnosing specific diseases. Furthermore, their versatility is hampered by the fact that they have been designed to record just one type of signal. Using ST-Qvar sensors, we acquired an electrocardiogram trace and single-channel scalp electroencephalogram from the frontal lobes, together with an electrooculogram. Excellent results from all three types of acquisition tests were obtained. The power consumption is very low, demonstrating that, thanks to the MEMS technology, a continuous acquisition is feasible for several days.

## 1. Introduction

Wearable technologies are starting to permeate multiple areas of life, including medical ones [1]. Longer monitoring windows and at-home supervision make wearables extremely appealing for clinicians, especially for all those exams still requiring obtrusive instrumentation and specific expertise for technical mounting. These are still strong limitations for the wide fruition of domestic recording through standard clinical equipment, even if portable. Among others, this is the case of electroencephalograms (EEGs) and electrocardiograms (ECGs), on which research is being heavily focused to provide wearable variants [2,3,4]. EEGs are usually performed by trained personnel in hospitals or clinics, placing a lot of electrodes all over the scalp, through long and uncomfortable setup procedures, connecting a lot of cables to bulky machines. However, this is not always the best way to obtain the desired information from the EEG. Indeed, in specific circumstances or for certain diseases, it would be necessary to monitor EEG continuously for several days in a domestic environment and free-living conditions. Although ambulatory EEG equipment is currently available for the long-term monitoring of brain activity in non-hospital settings, these devices are usually bulky and have a limited lifetime, presenting a conventional obtrusive setup comparable to the in-hospital arrangement (i.e., head cap with holes holding several electrodes on the scalp along with long wires connected to recording instrumentation). From these considerations comes the importance of a wearable technology able to provide both long-time and free-living monitoring, which are, in many cases, much more important than collecting many channels. This is the case, for example, for single-channel EEG research and applications, such as re-enabling communication through brain–computer interface approaches in patients suffering from advanced stages of paralyzing disorders, such as amyotrophic lateral sclerosis (ALS) [5]. Moreover, by providing real-time, long-term, and free-living dynamic information, new, noninvasive wearable solutions for EEG monitoring would support the differential diagnosis of pathological conditions characterized by transitory changes in focal brain activity, including epileptic and non-epileptic disorders, such as the loss of consciousness (i.e., cardiovascular syncope) and parasomnias (i.e., REM behavior disorder (RBD)) [6,7]. Lastly, a similar device would represent a feasible and practical tool also in hospital settings to continuously assess patients at the bedside, especially those suffering from specific pathological conditions, such as comatose survivors to cardiac arrest [8]. To testify the importance of portability, in the last few years, several wearable EEG devices have finally been introduced on the market. Unfortunately, the battery does not allow monitoring longer than half a day in any of them. Among the most popular, the system BitBrain Diadem is a 12-channel wireless headset recording EEG at 256 Hz for more than 8 h [9], while the system BrainBit is a 4-channel wireless headband recording EEG at 250 Hz for 12 h [10]. The company CGX has several distinct EEG headsets on the market with different features [11], used also for research purposes. In this frame, Li et al. studied age-related changes in cortical connectivity patterns during surgical anesthesia using the CGX 72-channels Mobile-Series, which offers 4 h battery life in Bluetooth mode or 10 h in local SD mode while acquiring EEG at 500 Hz [12]. Miller et al. studied the effects of computer-based brain training on cognitive performance on 21 employee volunteers using the CGX Quick 20 EEG headset, reporting 21 channels at a sampling frequency of 500 Hz and 6 h of battery life [13]. Kim et al. investigated movement intention classification through EEG signals using the CGX Quick 30, able to continuously acquire and transmit 32 channels for 8 h at 500 Hz [14]. In addition, we mention the company Wearable Sensing, which produces various EEG headsets, such as the DSI-7, which can acquire 7 EEG channels at 300 Hz for up to 12 h [15]. Mentalab produces a wearable system called Explore, which features an IMU and the possibility to acquire a 4- to 8-channel ExG at up to 1 kHz sampling frequency for 12 h maximum [16]. Finally, it is worth mentioning the EEG-based emotional valence detection performed by Apicella et al. with the AbMedica Helmate, a wireless, ultra-light EEG headband able to record at 512 Hz and transmit 8 EEG channels. Each trial lasted 30 s, but no information on the battery life is provided [17].

Looking at more revolutionary solutions, still at a research stage, Nakamura et al. recently proposed using in-ear EEG sensors [18,19]. Their device was based on a 64-channel NMIC front-end and a wireless transmission module. In Ref. [18], the authors reported a possible continuous usage for 44 h while streaming all 64 channels via Bluetooth, using a 550 mAh LiPo battery, which represents state-of-the-art power performance in the frame of research devices. Holle et al. realized a mobile ear-EEG, smartphone-based, to study auditory attention in everyday life. The system battery lasts for less than 7 h [20].

In general, ear-EEG ability to replace scalp EEG is still quite sub-par: indeed, since electrodes placement is limited to in-ear and around-ear, this family of EEG devices cannot be expected to reach the sensitivity of high-density cap-EEG in capturing brain-electrical activity, resulting in a significant loss of information [21].

Speaking of ECG, it is known that only extending monitoring for several days may allow to diagnose paroxysmal arrhythmias and evaluate potential arrhythmic symptoms and the mechanism of syncope [22]. ECG Holters, which usually acquire information for up to 48 h, often fail to identify heart rhythm disturbances [23], but manage to detect symptoms such as atrial fibrillation if the monitoring time is further prolonged for more than a few days [24]. The paper of Majumder et al. [25] reports on how many commercially available wearable systems are either limited in their recording sessions (the recording automatically stops after 30–300 s and has to be restarted) or, when the device does not show this limit, in their battery life, which does not allow more than 24 h of continuous recording. The authors’ own system is limited to 24 h by the adopted front end, as well as the very recent wearable ECG device Frontier X, whose nominal maximum recording window is 24 h [26].

It should be also noted that, at the present moment, the current processing power and battery life of wearables might constrain the upcoming use of sophisticated machine learning algorithms [27], which, on the contrary, forecast invaluable advantages unexplored to date. Finally, wearable technologies with lower power consumption would also enable to acquire, synchronously, more than one type of biopotential (i.e., polygraphic recording) for a long time, being compact and comfortable at the same time, and this would be a breakthrough for many applications in medicine. As an example, REM sleep classification is one of the cases which needs recording EEG activity, but also requires the synchronous acquisition of other biopotentials (e.g., ECG, electrooculogram (EOG), surface electromyography (sEMG)) for a whole night in the best patient condition, i.e., at home.

We can conclude that, despite the clinical importance of recording data for days or weeks, all the commercial wearable EEG and ECG devices show limited battery life, counting typically 12 h for EEG and 24 h for ECG. These performances are far from the days or weeks of monitoring originally envisioned [28], and hardly allow space for future developments. Indeed, it would be unfeasible to embed algorithms of deep learning and artificial intelligence directly on the boards, as well as to acquire more biopotentials along with any other physical or physiological parameter. Those limitations reduce the range of applicability of commercial devices. With such an overview, it is clear how new strategies, technologies, and solutions are necessary to reduce the power consumption of wearable sensing systems and improve their functionality and versatility.

We propose an MEMS multi-sensor system featuring a commercial charge transfer sensor (ST-Qvar), produced by STMicroelectronics and originally intended for presence tracking, to acquire an ECG trace and a single-channel EEG along with EOG. Specifically, we need to engineer the ST-Qvar hardware, design a dedicated front-end, and write a proper firmware to adapt the existing setup to polygraphic recording. Not only does charge transfer provide an incredibly low power, an unexplored solution to biopotentials acquisition, but it also enables adding this type of functionality to any existing MEMS. This means embedding even more functionalities to MEMS boards at near-zero additional power consumption (<20 μA per channel). For these reasons, ST-Qvars open up additional possibilities for wearable sensing and strengthening the role of MEMS technology in medical wearable applications for long-time synchronous acquisition of ECG, EEG, EOG, and inertial signals. To our knowledge, this is the first time that an electrostatic sensor based on electric charge transfer is used for this scope and, more generally, for health applications.

The paper is organized as follows: in Section 2 we describe the hardware components, the pre-amplifier circuit, and the firmware of a ST-Qvar board prototype. In Section 3, we show tests related to the acquisition of a single-channel EEG, the EOG, and the ECG and report on power consumption. For this stage of research, we performed tests on subjects in static conditions and compared with tests performed with standard equipment to prove the feasibility of the sensing technique. In Section 4, we discuss the viability of using ST-Qvar technology for long-time ECG, EEG, and EOG acquisitions, as well as other potential advantages of a dedicated system based on charge transfer.

## 2. Materials and Methods

The proposed system can operate recording data on an SD or transmit data wirelessly to an external elaborator. In principle, the recording operation mode does not present pitfalls and is the most convenient from the viewpoint of power consumption. For these reasons, in this section, we will focus on the operation mode which includes the wireless transmission of data via Bluetooth Low Energy, discussing the power consumption in this (more penalizing) case. The hardware setup described below is a prototype of the final miniaturized board, which STMicroelectronics is going to fabricate, assembled to demonstrate the performance of the ST-Qvar technology and the potential versatility of the system.

### 2.1. Hardware Setup

The hardware is made of a battery-powered sensing unit, made of multiple boards and equipped with electrodes. The sensing unit can record data on an SD or wirelessly transmit data to a laptop running a Bluetooth emulated virtual COM port on Matlab. In the latter case, Matlab receives data in real time and automatically stores the samples and the corresponding timestamps. The prototype is currently using an HM-19 Bluetooth Low Energy (BLE 5.0) module [29]. At present, this system is realized using general purpose hardware and its goal is testing the ST-Qvar feasibility in biopotentials acquisition. In imminent developments, we will realize an engineered, miniaturized, comfortable, and fully wearable dedicated system in collaboration with STMicroelectronics.

#### 2.1.1. Sensing Unit

**Figure 1:**
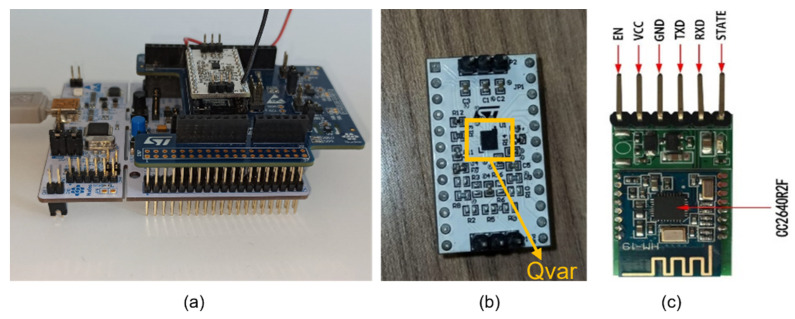
(**a**) The Nucleo L476RG + IKS01A1, (**b**) the ST-Qvar board, and (**c**) the Bluetooth module.

In this prototype, the sensing unit is made of an L053R8 Nucleo by STMicroelectronics, which embeds an ARM Cortex M0, along with an IKS01A1 X-Nucleo [30] mounted on top of it. The X-Nucleo features a 24-pin socket to host the ST-Qvar evaluation board. Being a prototype, the ST-Qvar board does not embed a microcontroller and needs this auxiliary setup for its use. The sensing unit is shown in Figure 1a. The ST-Qvar acquires with a maximum sampling frequency of 240 Hz. The microcontroller and the ST-Qvar communicate through standard I^2^C protocol once they are connected through the X-Nucleo.

#### 2.1.2. ST-Qvar Working Principle

Qvar, which stands for electric charge (Q) variation (var), is an electrostatic sensor that can be used for human presence and motion detection, touch detection, and user interface (UI) applications. In comparison with more established sensing techniques, such as those based on acoustic, resistive, capacitive, piezoelectric, optical, and electromagnetic principles, electrostatic sensors are relatively uncommon and less investigated. However, electrostatic sensors have clear advantages over other sensors, including cost-effectiveness and high sensitivity.

The ST-Qvar board, shown in Figure 1b, is an evaluation board, not yet commercial. It serves as a prototype to test the new triboelectricity-based charge transfer technology [31]. ST-Qvar can read the charge transfer occurring between the electrodes and the (moving) charged particles after friction between two materials. In general, an electrostatic charge is present on a material whenever it comes into contact with another material or a solid or liquid surface. The ST-Qvar sensor can read that charge with an extremely low current absorption, even lower than other sensors based on charge transfer.

The official STMicroelectronics application note AN755 available at [32] reports more information on ST-Qvar.

Given its very high sensitivity and its ability to measure these quasi-electrostatic potential changes, ST-Qvar is eligible for biopotentials acquisition, opening, for the first time, electrostatic sensors to health applications. In this case, however, the board needs some tweaking to the original polarization circuit to bring the biosignals levels in the input range of the onboard circuitry, as well as the addition of a pre-amplifier stage. These modifications will be discussed in the next section. ST-Qvar sensing channel architecture is made of an analog front-end (AFE), an analog to digital converter (ADC), a digital processing unit, and an SPI/I2C communication interface.

#### 2.1.3. Pre-Amplification Circuit

**Figure 2:**
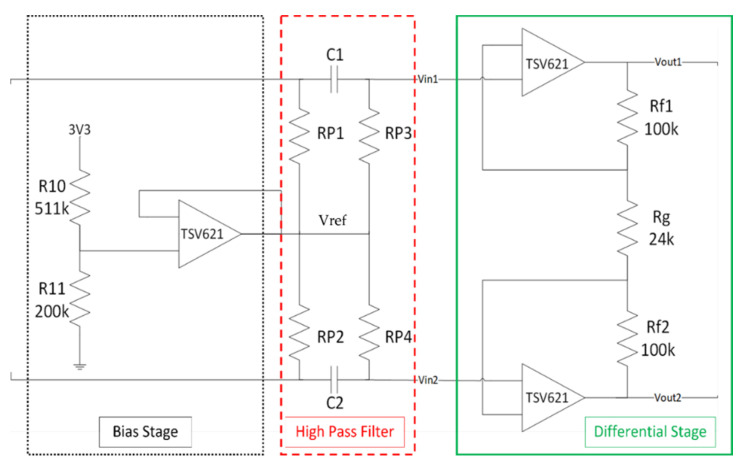
Pre-amplifier circuit.

Given the low amplitude of biopotentials, we had to design a proper front-end to amplify the signals before feeding them to the ST-Qvar. The chosen architecture for the pre-amplifier circuit is shown in Figure 2. The circuit is divided into three stages: the bias stage, highlighted with a black dotted outline, the high-pass filter stage, highlighted with a red dashed outline, and the differential stage, highlighted with a green continuous outline.

#### 2.1.4. Bias Stage

For its original purpose, ST-Qvar has to detect charge variations with high sensitivity. To accomplish this task, the producer originally designed the device with a DC supply of 3.3 V. However, we experimentally verified that waveforms with frequencies and amplitudes in our range of interest (0.5–100 Hz, 1 μV–10 mV) always produced saturated outputs. To avoid saturation, we had to replace the existing DC offset applied to the inputs with a lower one provided by the bias stage. In detail, we modified its external polarization to provide 0.9 V on each input by removing some voltage dividers on the PCB. The 0.9 V value is given by the bias stage shown in Figure 2. The bias stage is made of a voltage divider preceding an op-amp in voltage follower configuration. The voltage divider downscales the 3.3 V supply (V+) to the reference voltage (Vref) of 0.9 V:

$$\mathrm{Vref}=\frac{3.3\;V\times 200\;kΩ}{200\;kΩ+511\;kΩ}=0.9\;V$$

The signal is then fed to a voltage follower, used as a buffer to separate the voltage divider from the rest of the circuit.

#### 2.1.5. High-Pass Filter

**Figure 3:**
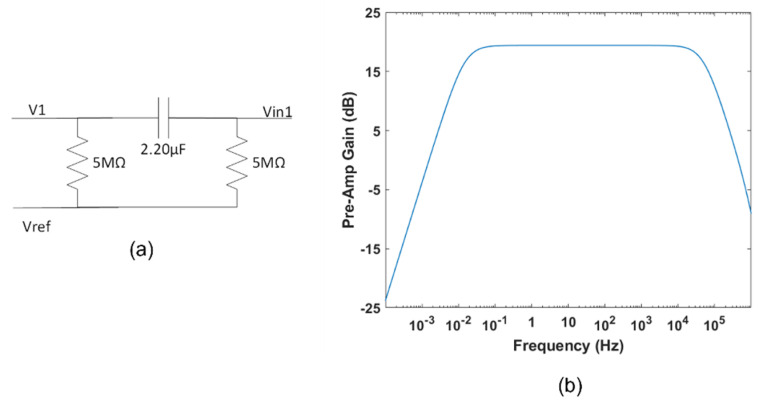
(**a**) The detailed high−pass filter; (**b**) gain vs. frequency.

In addition to the bias stage, we introduced a high-pass filter, shown in Figure 2 and detailed in Figure 3. This filter is necessary to eliminate DC and electrodes offset. Our first design criterion was to provide a high-input impedance to the pre-amp stage, thus allowing the system to properly read the signals of interest. We experimentally verified that a resistor of 5 MΩ is high enough to achieve this requirement. Second, we had to choose a capacitance value to ensure a cutoff frequency well below the lower bound of our useful bandwidth (around 0.5 Hz; see, for example, [33]). Consequently, we adopted a capacitor of 2.20 μF, obtaining a cut-off frequency f_p1_ of 14.4 mHz. Finally, LT-Spice simulations highlighted that the second pole frequency f_p2_ falls well beyond the bandwidth upper limit, as shown in Figure 3b.

$$f_{p1}=\frac{1}{2{\pi R}_{P3}C_{1}}=14.4\;\mathrm{mHz}$$

$$f_{p2}=54\;\mathrm{kHz}$$

#### 2.1.6. Fully Differential Stage

**Figure 4:**
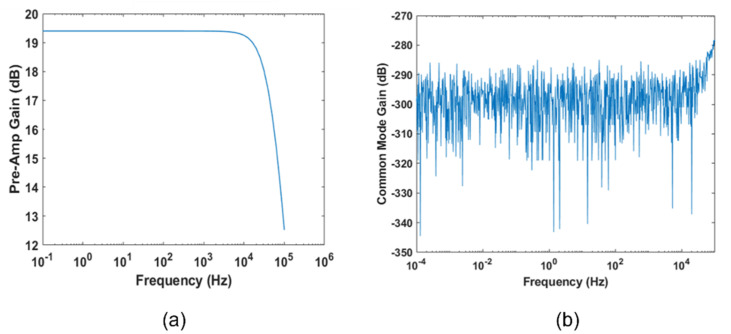
Simulations of the differential stage performed on LT Spice: (**a**) Differential gain; (**b**) common−mode gain.

As a final stage, we introduced a fully differential amplifier. It needs to reject the common mode signal and to amplify the difference between the two inputs. The Qvar linear gain is 78, so we did not need to significantly increase the front-end differential gain, but rather to properly attenuate the common mode gain. Thus, our design criterion was to obtain a common mode rejection ratio (CMRR) higher than 300 dB. With the aid of LT-Spice simulations, we opted for the following choices. Setting the resistors values as reported in Figure 2, we obtain the differential gain, Ad (Figure 4a):

$$\mathrm{Ad}=1+\frac{\mathrm{Rf}1}{\mathrm{Rg}}+\frac{\mathrm{Rf}2}{\mathrm{Rg}}=9.33=19.4\;\mathrm{dB}$$

Common-mode gain simulation results are shown in Figure 4b. Thus, the common-mode rejection ratio is

$$\mathrm{CMRR}\;\left(\mathrm{dB}\right)={\;A}_{d}^{\mathrm{dB}}-A_{\mathrm{cm}}^{\mathrm{dB}}=19.4\;\mathrm{dB}-\left(-300\right)\;\mathrm{dB}=319.4\;\mathrm{dB}$$

#### 2.1.7. Noise Simulations

One of the main problems in this kind of system is the high impedance of the skin, which worsens when using dry electrodes, adopted in many commercial devices. That is why, in our tests, we used wet electrodes, although they are less comfortable. In this case, the impedance mismatch of the two electrodes is a much bigger problem and, for this reason, we perform noise simulations. Anyway, with our current scenario being mostly static (as, for example, sleep monitoring), dry electrodes would be particularly disadvantaging since they could not benefit of increased conductivity due to sweating, as happens for sport-targeted devices. It is clear how the high-value resistors (used to realize the body polarization) and the op-amps represent the main noise sources. That is why, in the following, we only consider those two sources. For the noise analysis, we replaced each resistor of the designed front-end (Figure 2) with its equivalent noise model.

The input source can be modeled as a differential voltage generator between the two electrodes on the patient skin, so in this case, the deactivation of the voltage generator has to be conducted in a specific manner. We performed the noise evaluations considering two principal cases of the skin–electrode impedance:

1. Z_ep_ = ∞.
2. Z_ep_ = 0.

**Figure 5:**
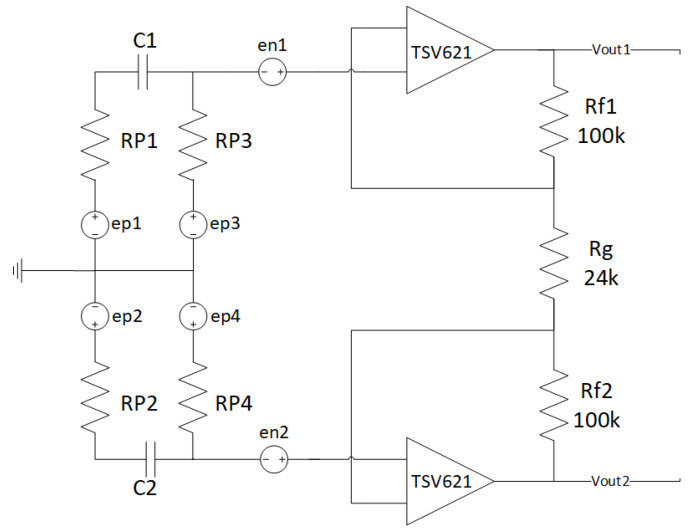
Noise analysis of the proposed pre-amp stage with Zep = ∞.

Case 1 (Z_ep_ = ∞) is depicted in Figure 5. The two main sources of noise are the bias resistors and the op-amps. The average mean square values of the op-amps output noise are given by

$$E_{\mathrm{diff}}^{2}=\int \left[e_{n}^{2}\left(1+\frac{R_{f1}}{R_{g}}+\frac{R_{f2}}{R_{g}}\right)\right]\mathrm{df}$$

where the term inside the round brackets is the pre-amplifier gain (A_d_ = 9.33) and e_n_ is the input noise voltage of the TSV62XA op-amp, which equals 77 nV/$\sqrt{\mathrm{Hz}}$.

The average mean square values of the bias resistors output noise are given by

$$E_{\mathrm{res}}^{2}=\int \left[e_{p}^{2}\left(\frac{R_{P}}{2R_{P}}\right)\left(1+\frac{R_{f1}}{R_{g}}+\frac{R_{f2}}{R_{g}}\right)\right]\mathrm{df}$$

where $e_{p}^{2}=4K_{B}\mathrm{TR}$ is the Johnson noise, K_B_ is the Boltzmann’s constant, T is the resistor absolute temperature, and R is the resistor value. Considering four bias resistors and two op-amps, the total output noise of the pre-amp circuit is given by

$$E_{\mathrm{TOT}}=\sqrt{E_{\mathrm{diff}1}^{2}+E_{\mathrm{diff}2}^{2}+E_{\mathrm{res}1}^{2}+E_{\mathrm{res}2}^{2}+E_{\mathrm{res}3}^{2}+E_{\mathrm{res}4}^{2}}$$

Solving all integrals in the bandwidth 0.5 ÷ 40 Hz, we obtain

$$E_{\mathrm{input}\_\mathrm{TOT}}=\frac{E_{\mathrm{TOT}}}{A_{d}}=1.68{\mathrm{uV}}_{\mathrm{rms}}$$

**Figure 6:**
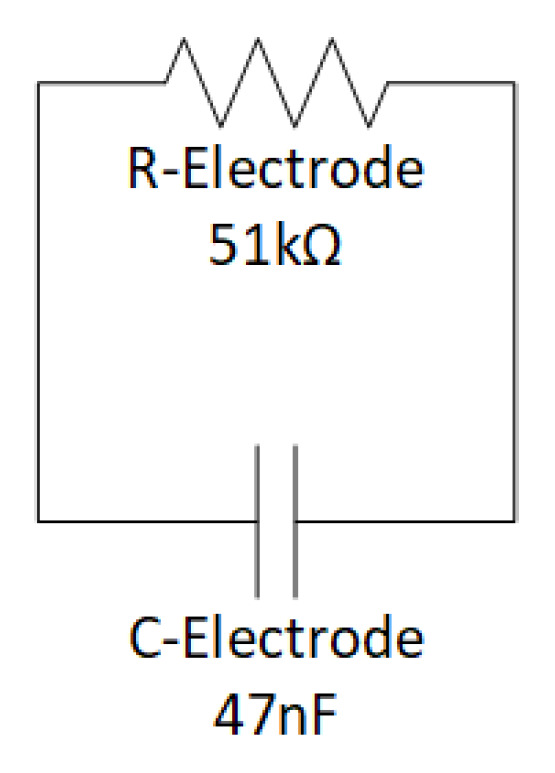
Electrode model according to IEC 60601–2–47 standard.

**Figure 7:**
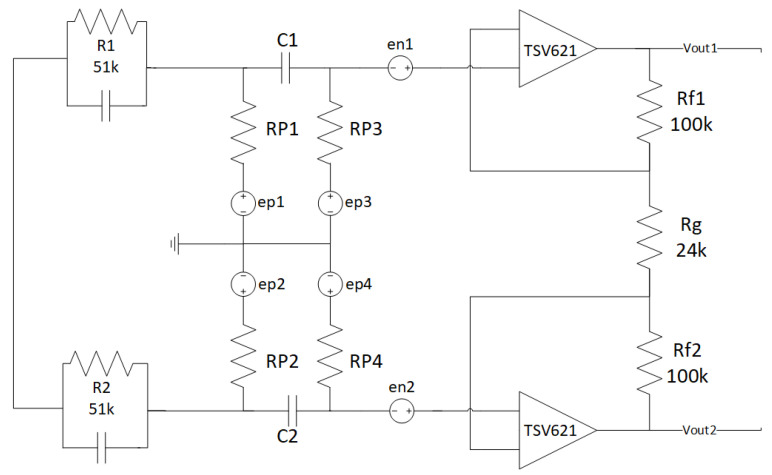
Noise analysis of the proposed pre-amp stage with Zep = 0.

We consider the equivalent electrode skin impedance Z_ep_ established by the standard IEC 60601-2-47 that concerns the basic safety and essential performance of ambulatory electrocardiographic systems [34]. In this case, disabling the two inputs is equivalent to short-circuiting the input terminals. Thus, the simulation is developed by modeling the electrode–skin impedance as the parallel of one 51 kΩ resistor and one 47 nF capacitor, as shown in Figure 6. Case 2 (Z_ep_ = 0) is shown in Figure 7.

The average mean square values of the output noise depending on the op-amps are equal to the previous case.

Considering the A node and B node, the total output voltage noise of the pre-amp circuit is

$$E_{\mathrm{TOT}}=\sqrt{[2\left(e_{n}^{2}A_{d}^{2}\right)+\left(A_{d}V_{\mathrm{AB}}\left(f\right){)}^{2}\right]B}=\mathrm{Ad}\times 0.56{\;µV}_{\mathrm{rms}}$$

The input referred noise is found by dividing E_TOT_ by the pre-amplifier gain Ad, obtaining

$$E_{\mathrm{input}\_\mathrm{TOT}}=\frac{E_{\mathrm{TOT}}}{\mathrm{Ad}}=0.56{\;µV}_{\mathrm{rms}}$$

**Figure 8:**
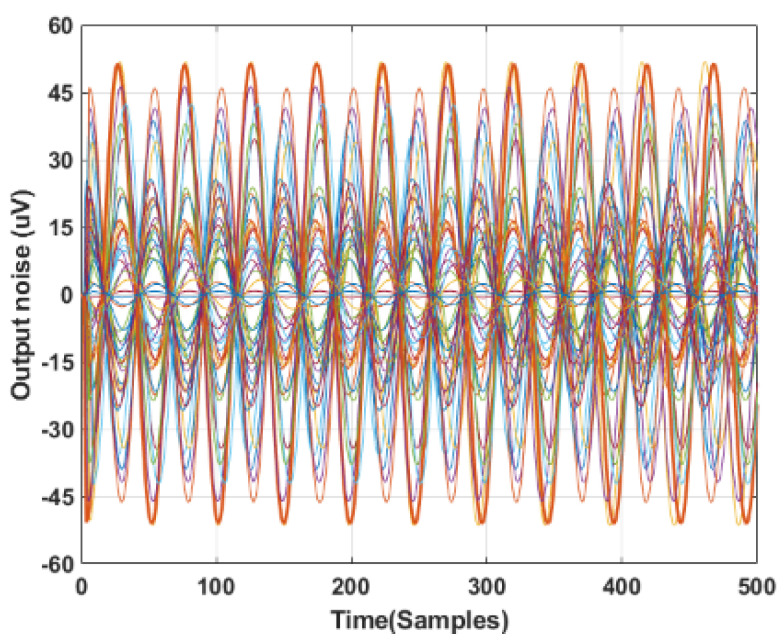
Monte Carlo simulations on pre−amplifier stage with 1% passive tolerance.

Finally, in a specific circumstance where the battery is not available, a cabled power supply is viable. In this case, it is worth providing some information on the common-mode noise introduced by the components (electrodes included) mismatching. A common-mode signal, with an amplitude of 1 V and a frequency of 50 Hz, has been sent to the inputs of the pre-amplification circuit (the differential signal is not considered for this analysis). Considering the balanced circuit in Figure 2, the common-mode output is massively reduced, with an Acm of −300 dB (as shown in Figure 4b). It is important to understand which values of the passive components lead to an increase in the common-mode output. We used the Monte Carlo method to evaluate the common-mode output, considering various combinations of all the passive components. We performed simulations with LT Spice 17.0.32.0, considering a tolerance in the maximum range of 1% for the passive components. Results are shown in Figure 8, where different colors indicate different combinations of resistance and capacitor values in that range. The worst case of the simulation shows a peak-to-peak amplitude of 100 μV.

#### 2.1.8. HM-19 Bluetooth Low Energy Module

At the current stage, for the prototype under study, we opted for the HM-19 BLE module embedding a BLE CC2640R2F chip from Texas Instruments [29], which is particularly easy to set up. It allows to broadcast via Bluetooth anything sent on the Nucleo serial ports, such as UARTs. It can be set in Master or Slave mode and its parameters (such as name, password, baud rate, etc.) can be easily configured. The module allows communication between a microcontroller, such as the Cortex M0 embedded in the Nucleo board, and a device equipped with Bluetooth communication (PC, smartphone, or tablet). We connected the TXD and RXD pins of the HM-19 module to the L053R8 UART-1 pins and set the bit rates of both to 57,600 bit/s. HM-19 can work with a 3.3 V supply. It is shown in Figure 1c. In the future, the dedicated boards will mount a BLE by STMicroelectronics.

#### 2.1.9. Receiver Unit

**Figure 9:**
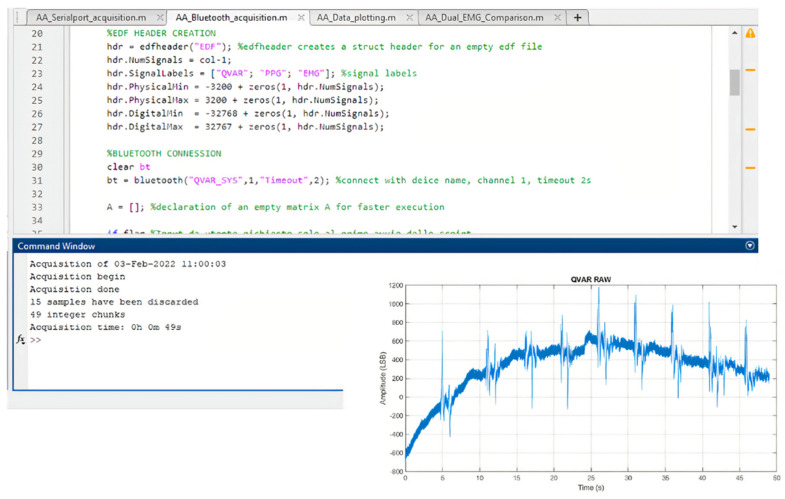
Matlab application interface for data acquisition.

The transmitter unit is connected via BLE to a laptop running Matlab with BLE toolbox, which acts as the receiver unit. The BLE toolbox allows reading an incoming Bluetooth transmission by setting a virtual COM port and treating the Bluetooth link as a standard serial transmission. In this way, data incoming from the ST-Qvar sensors are received in real time and automatically saved at the end of the recording. Date, time, recording duration, and the number of acquired samples are saved. Matlab waits for the Bluetooth pairing between the HM-19 and the laptop, then starts acquiring data from the Bluetooth virtual COM port. The Matlab interface is shown in Figure 9.

### 2.2. Firmware Description

**Figure 10:**
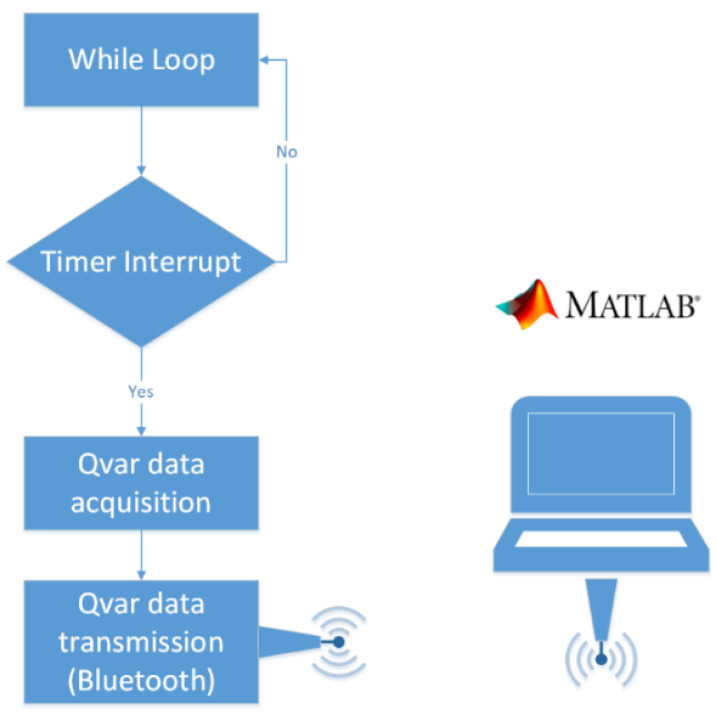
Block diagram of the proposed firmware. The overall system clock is 3 MHz and the interrupt clock is 240 Hz.

In our prototype, ST-Qvar acquisition is handled by the Cortex M0 microcontroller embedded on the Nucleo L053R8 board. We wrote the firmware with Keil μVision V5 software. Our first criterion was to acquire signals at the maximum sampling frequency of the ST-Qvar (240 Hz). Second, we wanted to acquire through an interrupt routine. As a last criterion, our interrupt had to be triggered by a timer. Figure 10 shows the logical scheme of the implemented firmware. On each timer tick, the microcontroller acquires the charge value via I^2^C from the ST-Qvar and sends it to the Bluetooth transmitter via serial port. The timer is set to tick at 240 Hz. The BLE transmits the data directly to a laptop running Matlab.

## 3. Tests and Discussion

Tests discussed in this section have been performed on subjects in static conditions. Obviously, motion can introduce additional practical difficulties, noise sources, and artifacts. However, at the current stage of the research, the target is to prove the feasibility of the sensing technique. After proving the concept in static conditions, tests on moving subjects will be a natural extension. A single ST-Qvar sensor was used to separately acquire an ECG trace anda single-channel EEG (concurrently with an EOG if scalp electrodes are placed on the frontal lobes). The number of ST-Qvar sensors integrated into the board determines the number of biopotentials which can be detected, as well as the number of EEG channels. With the current absorption of a ST-Qvar sensor being in the order of twenty microamperes and its size being in the order of 4 mm^2^, integration of a few ST-Qvar sensors is not penalizing from the viewpoint of the system performance and wearability. Tests were performed on five healthy volunteers of age 25–35, using standard AgCl electrodes with size 50 × 48 mm. Electrode positioning, skin cleaning, tests setup, and execution were supervised by doctors, who also examined the test results.

### 3.1. ECG Test: Qvar vs. Gold Standard

**Figure 11:**
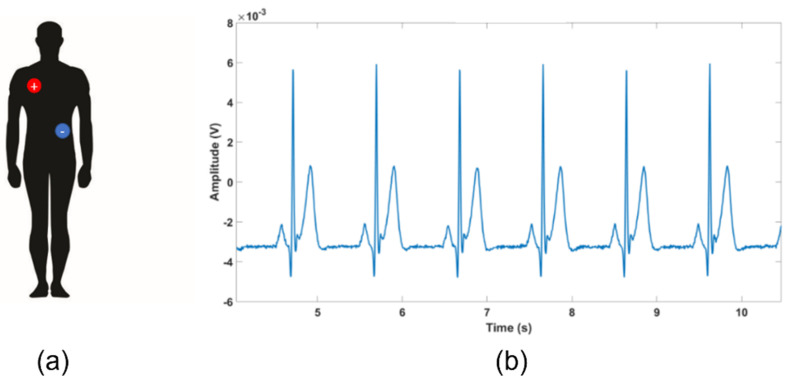
(**a**) Electrodes positioning; (**b**) typical ECG raw trace.

Concerning the ST-Qvar setup in the ECG signal, we used a single-lead ECG to estimate the heart frequency through the detection of the R-peaks and the evaluation of RR intervals. More in detail, we adopted a lead D1 configuration implying the positive and negative electrodes placed on the left and right upper extremities, respectively. For each subject, we positioned wet commercial Ag/Cl electrodes in RA-LL configuration, as shown in Figure 11a [35,36]. The acquired raw ECG trace from one of the candidates is displayed in Figure 11b.

**Figure 12:**
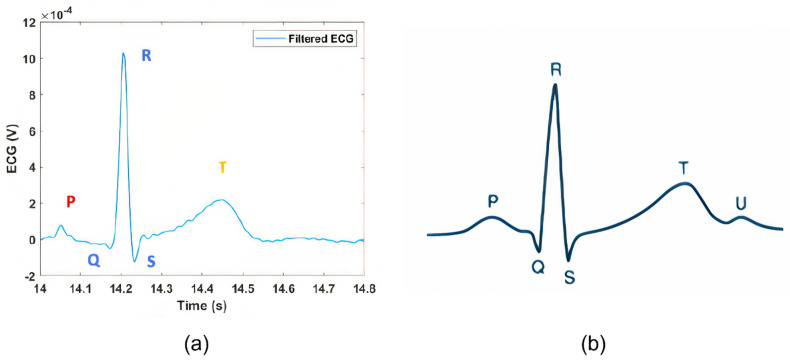
(**a**) Filtered ST−Qvar ECG: single oscillation; (**b**) Typical ECG, single oscillation.

A single ECG oscillation from Figure 11 is zoomed-in in Figure 12a, where it is possible to recognize all the typical features of ECG, namely, the P wave, the QRS peak, and the T wave [37]. For comparison, a typical ECG waveform is shown in Figure 12b [38]. Figure 12. Table 1 reports the obtained mean values for the main ECG features, calculated from the five-subjects population.

**Figure 13:**
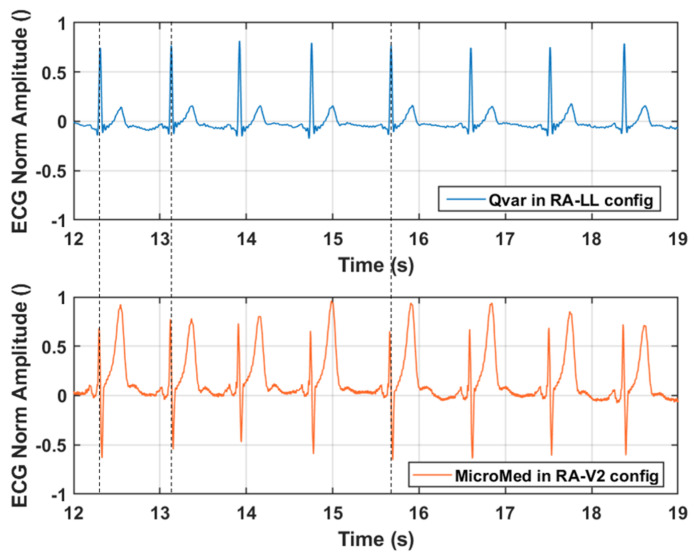
ECG trace acquired simultaneously from Qvar and MicroMed systems, from different derivations. Dashed lines show the R−peaks correspondence between the two tests.

In conclusion, in Figure 13 we report a fraction of a simultaneous two minutes’ acquisition of ECG signal performed by Qvar and MicroMed systems. In multi-channel ECG acquisitions, different electrodes locations change the morphology of the ECG waveform, but the R-peak period, and thus the RR interval, does not depend on the chosen derivation. This happens because heart rate measurement comes from RR interval and must be derivation independent. To prove the robustness of our sensing technique, also taking this into account, we kept the configuration from Figure 11a for the ST-Qvar system and adopted a different one for the gold standard. In particular, we placed the MicroMed electrodes in RA-V2 configuration [36].

Looking at Figure 13, it is clear how the R-peaks from the Qvar system are perfectly synchronized with the ones from the gold standard, as expected even with different derivations. Black dashed vertical lines are reported for convenience. Based on the use of a single-lead ECG, our approach would be suitable for a basic heart monitoring of rhythms to detect arrhythmias, such as atrial fibrillation, as well as to examine heart rate variability during specific sleep stages, as previously reported [39,40].

### 3.2. EEG Test: ST-Qvar vs. Gold Standard

**Figure 14:**
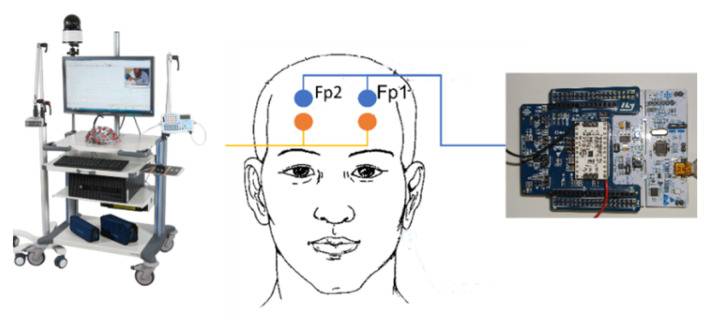
Electrodes positioning for EEG–EOG test for MicroMed (**left, orange electrodes**) and ST-Qvar (**right, blue electrodes**).

Under the supervision of neurologists, a single subject underwent an acquisition of frontal single-channel EEG with ST-Qvar and the MicroMed Medical device (again considered as our gold standard) simultaneously. Following the 10–20 International System guidelines, we placed the electrodes in locations Fp1–Fp2, as shown in Figure 14. The electrodes of the MicroMed and the electrodes of the ST-Qvar were not overlapped but were as close as possible to each other. With this setup, we asked the subject to simply keep their eyes closed for few seconds to record EEG activity.

**Figure 15:**
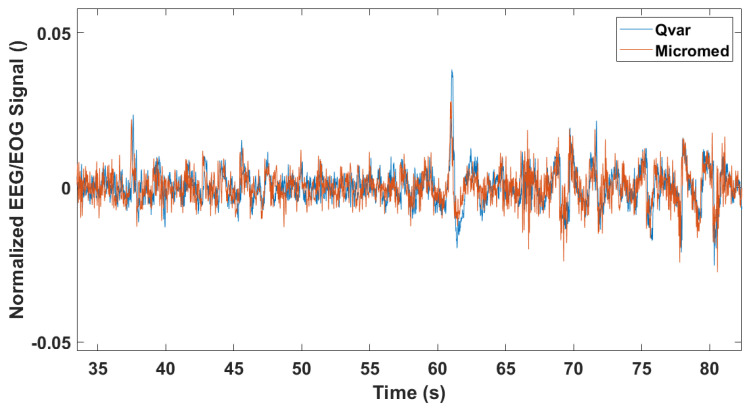
MicroMed vs. ST−Qvar raw traces from frontal single-channel EEG acquisition.

This test served as a general indication of the ST-Qvar’s ability to record a comparable signal with respect to the gold standard. With this being a single channel acquisition from Fp1–Fp2 electrodes, the only analysis performed is a time domain comparison. Results are shown in Figure 15, where we report only a fraction of the two raw signals. The two traces collected with ST-Qvar and MicroMed match significantly. The minimal amplitude difference is due to the slightly different positions of the electrodes in the two cases.

**Figure 16:**
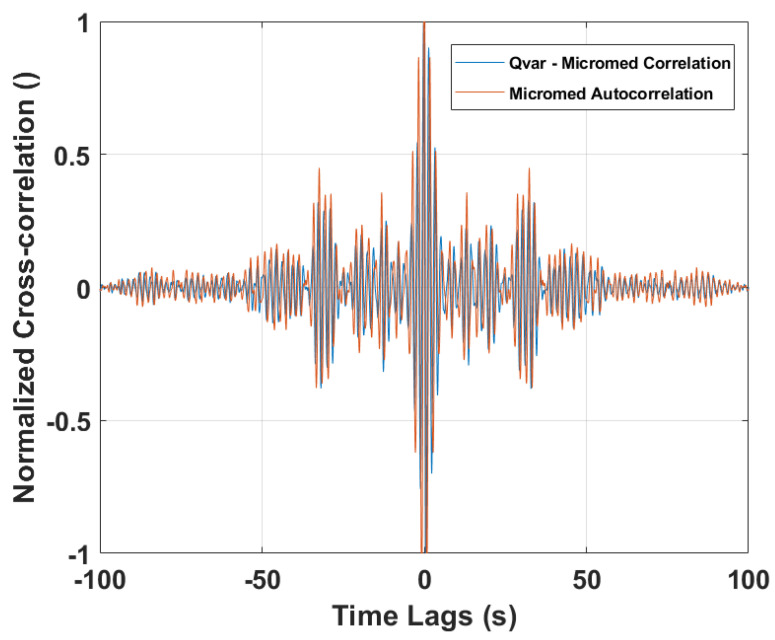
Cross-correlation between ST−Qvar and MicroMed acquisitions. MicroMed signal autocorrelation.

Two correlations were performed on those (entire) signals and are shown in Figure 16. In particular, the autocorrelation of the gold standard is plotted together with the cross-correlation of the ST-Qvar one, with respect to it. As is clear from the graph, the two traces are overlapped. Furthermore, both traces have their maximum at zero lag.

### 3.3. Rapid Eye Movement Detection Test (EOG): ST-Qvar vs. Gold Standard

**Figure 17:**
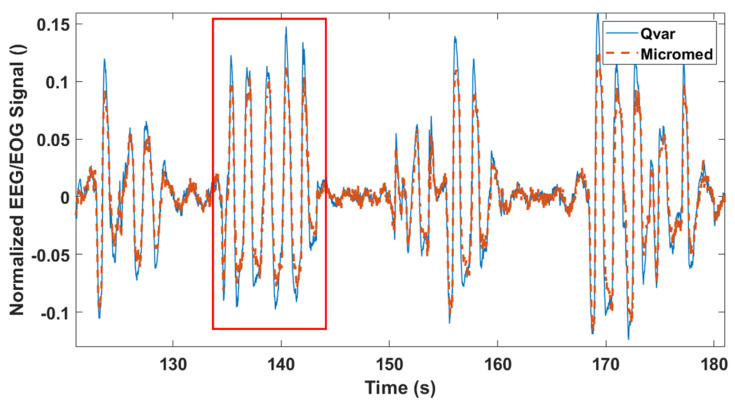
EOG trace recorded from ST−Qvar and the gold standard during clockwise eye movements. A single window of the eye movements is highlighted with a rectangle.

**Figure 18:**
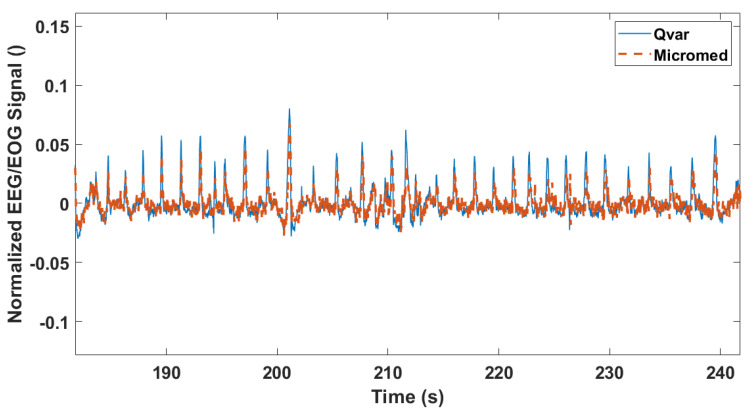
EOG trace recorded from ST−Qvar and the gold standard during eye blinking test. Each peak represents an eye blink.

One of the main artifacts of frontal channel EEG is the electrooculogram [41,42]. Despite being a high-amplitude artifact in EEG recordings, EOG is a useful signal for many applications [43] and diagnoses [44] because it allows to detect eye movements. One of the main applications of EOG regards the classification of sleep stages through the evaluation of several physiological variables, including eye movements [45]. More in detail, to identify the so-called “rapid eye movement (REM) sleep”, it is necessary to record a random rapid movement of the eyes in a range of directions. Accordingly, our test features clockwise eye movements to verify the ability of our system to recognize multidirectional eye movements by EOG signals. Following the International 10–20 System Standard [46], electrode positioning in the Fp1 and Fp2 locations allows recording the EOG. MicroMed electrodes were also placed in Fp1 and Fp2 configuration, very close to the ST-Qvar ones. The subject was asked to rotate eyes (closed) clockwise for some seconds, then to pause and repeat this protocol four times. Results are shown in Figure 17. High-amplitude oscillations (highlighted with a red continuous rectangle) represent eye movements and can be easily recognized with respect to the background. After the eye movements, the subject was also asked to blink their eyes repeatedly. This test is shown in Figure 18. Even in this case, the signals from ST-Qvar and the gold standard perfectly overlap. All these elements point to the accuracy of the ST-Qvar recordings with respect to the gold standard, significantly supporting the feasibility of charge variation technology for biopotentials acquisition.

### 3.4. Power Consumption

In this paragraph, we report values of current absorption experimentally measured in the laboratory by a digital multimeter and a source measurement unit (SMU). In operating mode, the entire system current absorption is approximately 4.4 mA. In detail, the microcontroller draws 2 mA (the Nucleo board in total draws 4 mA, due to an LED always in the ON state), while the ST-Qvar sensor and the pre-amplifier circuit draw, respectively, 20 μA and 180 μA. The Bluetooth Low Energy module draws 2.6 mA. On this basis, the expected life of a conventional Li battery for light wearable applications (3.7 V, 500 mAh) is of 227 h (9.5 days) in the recording operation mode and 104 h (4.3 days) in the wireless transmission operation mode. In a hypothetical ST-Qvar multi-channel system and in the worst-case scenario of a dedicated pre-amp for each ST-Qvar, every additional channel would increase the current absorption by 200 μA, corresponding to a reduction of 18 h with a 500 mAh battery. Using two AA 1000 mAh batteries, similar to most of the devices on the market, the autonomy would increase by a factor of four with respect to our current implementation. A recap of the ST-Qvar system performance is provided in Table 2. The hypothetical 30-channel ST-Qvar system is also included in the table, just to highlight that, also in a multi-channel case, this system would be competitive with commercial ones in terms of battery life. Additional optimizations to the pre-amp are also possible to further reduce the value of a 200 μA/channel.

## 4. Discussion

In the previous sections, we saw that each ST-Qvar sensor, with electrodes connected to the inputs, enables a single channel biopotential acquisition. The current absorption of a single ST-Qvar sensor is 20 μA and its size is in the order of 4 mm^2^. Thus, integration of one or more ST-Qvar sensors in an MEMS board would improve the board functionality by enabling single/multi-channel ECG, EEG, or EOG acquisition without significantly penalizing battery life and wearability. Therefore, the ST-Qvar sensor technology opens not only for more power-efficient and accurate dedicated systems, but also for easy single/multiple channel biopotential acquisition (polygraphic acquisition) in general-purpose hardware at near-zero additional cost. This would be important for several clinical prospects, including practical applications in both extra- and in-hospital settings for the recognition and monitoring of different pathological conditions. As an example, the proposed wearable system would be a valuable support for innovative approaches of the brain–computer interface in paralyzing diseases (e.g., amyotrophic lateral sclerosis), as well as for the differential diagnosis of neurological conditions presenting overlapping clinical symptoms, such as some epileptic and non-epileptic disorders (e.g., syncope and REM behavior disorders).

In our tests, we used a prototype board with a single ST-Qvar sensor to demonstrate the feasibility of recording an ECG trace and, in the alternative, a single-channel EEG associated with EOG from subjects in static conditions. As a result, we obtained an excellent agreement between ST-Qvar and laboratory gold standard, thus confirming the validity of the approach. Obviously, in non-static scenarios, motion of subjects can introduce additional practical difficulties, noise sources, and artifacts. However, as already stated, at the current stage of the research, the target is proving the feasibility of the sensing technique. To our knowledge, this is the first time that an electrostatic sensor based on electric charge transfer is used for this scope and, more generally, for health applications. Thus, we first focused on applications involving static situations where long-time polygraphic acquisition is of outmost importance, such as in sleep monitoring, where several biopotentials (beta rhythms of EEG, ECG, EOG, and others) need to be acquired synchronously.

After that, proving the concept also in moving subjects will be a due extension.

Tests highlighted a potential battery life of up to 9 days with a 500 mAh battery, which is a smaller supply than the two AA batteries that many commercial systems use. The microcontroller is an unoptimized part of the system, so it accounts for the biggest part of the consumption. On the contrary, with its low absorption of only 20 μA, the ST-Qvar sensor approximately takes 1% of the total system current absorption. Together with the 180 μA of the pre-amp (which has further room for engineering), this represents an extremely-low-power front-end for ECG/EEG, especially since, in a dedicated architecture, the same pre-amp could serve more channels within a bandwidth/power tradeoff.

As the opposite, all the commercial wearable EEG and ECG devices show performances far from the days or weeks of monitoring originally envisioned, and hardly allow space for future developments. Indeed, with those performances, it would be unfeasible to embed algorithms of deep learning and artificial intelligence directly on the boards, as well as to acquire more biopotentials along with any other physical or physiological parameter.

## 5. Conclusions

In this work, we investigated the feasibility of biopotential recording by the means of charge transfer sensing technique. We used an off-the-shelf device (ST-Qvar by STMicroelectronics), and, for the specific target, we engineered its hardware, designed a dedicated front-end and wrote a proper firmware thus enabling acquisition of ECG, EEG, EOG traces. To our knowledge, using an electrostatic sensor based on electric charge transfer for this scope and, more generally, for health applications is an innovative approach. Tests were supervised by neurologists and were performed with our device and a laboratory gold standard, simultaneously. The excellent agreement between ST-Qvar and gold standard confirmed the validity of the approach. In comparison with more established sensing techniques, such as those based on acoustic, resistive, capacitive, piezoelectric, optical, and electromagnetic principles, electrostatic sensors are relatively uncommon and less investigated. However, they have clear advantages over other sensors, including cost-effectiveness and high sensitivity. Their realization in MEMS technology also ensures extremely low power consumption and enables adding this type of functionality to any existing MEMS (inertial sensors, for example). This means that in principle it is possible to embed even more functionalities to MEMS boards at near-zero additional invasivity, expense, and power consumption. ST-Qvars are open to additional possibilities for wearable sensing and strengthening the role of MEMS technology in medical wearable applications for long-time domestic monitoring.

## Figures and Tables

**Table 1:** ECG time intervals and amplitude ratio: nominal vs. ST-Qvar values.

ECG Feature	Nominal	ST-Qvar (Mean Value)
PR Interval	0.12 ÷ 0.2 s	0.16 s
QRS Interval	0.06 ÷ 0.1 s	0.06 s
QT Interval	0.42 s	0.38 s
QRS Amplitude	>0.5 mV	1 mV

**Table 2:** ST-Qvar prototype consumption recap.

Component	Current Absorption (mA)	500 mAh Battery Life (h)	2 AA 1000 mAh Battery Life (h)
Cortex M0	2		
ST-Qvar Sensor	0.02		
Pre-amplifier Stage	0.18		
BLE module (optional)	(2.6)		
Total system (1 channel)	2.2 (4.8 with BLE)	227 (104.2 with BLE)	908 (416 with BLE)
Hypothetical 30-channels	8.2 (10.8 with BLE)	61 (46 with BLE)	243 (185 with BLE)

## Data Availability

The data presented in this study have been recorded from healthy subjects. Although they gave their consent to show the obtained results, their raw and partial/complete ECG/EEG recordings are covered by privacy.
